# Anévrisme de l’artère iliaque primitive droite

**DOI:** 10.11604/pamj.2018.30.106.15751

**Published:** 2018-06-11

**Authors:** Mohamed Jira, Taoufik Amezyane

**Affiliations:** 1 Hôpital Militaire d’Instruction Mohammed V, Service de Médecine Interne, Rabat, Maroc

**Keywords:** Anévrisme, artère iliaque primitive, maladie de Behçet, Aneurysm, common iliac artery, Behcet’s disease

## Abstract

We here report the case of a 55-year old patient, with a history of recurrent buccal aphthosis, hospitalized for pelvic pain of sudden onset. Clinical examination showed sensitive pulsatile pelvic mass with blowing sound suggesting vascular origin. Examination of external genitalia showed scrotal scar after healing of aphthous ulcers. Laboratory test showed inflammatory syndrome with CRP levels of 80 mg/L, syphilis serological test was negative, doppler ultrasonography revealed primary aneurysm of the right common iliac artery and arteriography confirmed the diagnosis. The diagnosis of Behcet’s disease was retained because of bipolar aphthosis and vascular involvement. Treatment was based on surgical resection of the aneurysm, bolus corticosteroids for three days, then oral corticosteroids and monthly intravenous cyclophosphamide infusion. Patient’s outcome was favorable.

## Image en médecine

Il s'agissait d'un patient âgé de 55 ans, ses antécédents étaient marqués par une aphtose buccale récidivante, il était hospitalisé pour une douleur pelvienne d'installation brutale et chez qui, l'examen clinique trouvait une masse pelvienne sensible, pulsatile et soufflante à l'auscultation évoquant une origine vasculaire, l'examen des organes génitaux externes trouvait une cicatrice d'aphtose scrotale. Le bilan biologique objectivait un syndrome inflammatoire avec CRP à 80 mg/l, la sérologie syphilitique était négative, l'écho-doopler pelvienne était faveur d'un anévrisme de l'artère iliaque primitive droite et l'artériographie confirmait le diagnostic Le diagnostic de la maladie de Behçet était retenu devant l'aphtose bipolaire et l'atteinte vasculaire. Le traitement comportait une exérèse chirurgicale de l'anévrisme, corticothérapie en bolus de trois jours puis en relai par voie orale et une perfusion mensuelle de cyclophosphamide. L'évolution était favorable.

**Figure 1:**
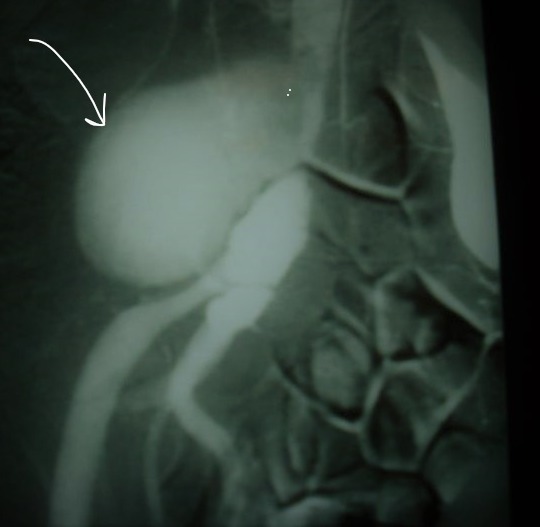
L’artériographie montrait un anévrisme de l’artère iliaque primitive droite

